# Thulium laser transurethral vaporesection of the prostate versus transurethral resection of the prostate for men with lower urinary tract symptoms or urinary retention (UNBLOCS): a randomised controlled trial

**DOI:** 10.1016/S0140-6736(20)30537-7

**Published:** 2020-07-04

**Authors:** Hashim Hashim, Jo Worthington, Paul Abrams, Grace Young, Hilary Taylor, Sian M Noble, Sara T Brookes, Nikki Cotterill, Tobias Page, K Satchi Swami, J Athene Lane, UNBLOCS Trial Group, Rupert Beck, Christopher Blake, Kim Davenport, Oliver Kayes, Jonathan Sullivan, Nicholas Cohen, Mathialagan Murugesan, Anthony Timoney, Lyndsey Johnson, Benita Adams, Angela Allan, Carol Brain, Fiona Hammonds, Joan Henderson, Paula Hilltout, Bernadette Kilbane, Leigh Morrison, Wendy Robson, Lorraine Wiseman, Vivian Zinyemba, Chris Metcalfe, Rafiyah Khan, Alan Uren, Aideen Ahern, Aida Moure Fernandez, Barbara Warnes, Tom Steuart-Feilding, Christopher Pawsey, Julie Plant, Mai Baquedano, David Carmichael

**Affiliations:** a Bristol Urological Institute, Southmead Hospital, North Bristol NHS Trust, Bristol, UK; b Bristol Randomised Trials Collaboration, Bristol Trials Centre, University of Bristol, Bristol, UK; c Department of Urology, Freeman Hospital, The Newcastle upon Tyne Hospitals NHS Foundation Trust, Newcastle upon Tyne, UK; d Urology Department, Aberdeen Royal Infirmary, NHS Grampian, Aberdeen, UK

## Abstract

**Background:**

Transurethral resection of the prostate (TURP) is the standard operation for benign prostatic obstruction. Thulium laser transurethral vaporesection of the prostate (ThuVARP) is a technique with suggested advantages over TURP, including reduced complications and hospital stay. We aimed to investigate TURP versus ThuVARP in men with lower urinary tract symptoms or urinary retention secondary to benign prostatic obstruction.

**Methods:**

In this randomised, blinded, parallel-group, pragmatic equivalence trial, men in seven UK hospitals with bothersome lower urinary tract symptoms or urinary retention secondary to benign prostatic obstruction were randomly assigned (1:1) at the point of surgery to receive ThuVARP or TURP. Patients were masked until follow-up completion. Centres used their usual TURP procedure (monopolar or bipolar). All trial surgeons underwent training on the ThuVARP technique. Co-primary outcomes were maximum urinary flow rate (Qmax) and International Prostate Symptom Score (IPSS) at 12-months post-surgery. Equivalence was defined as a difference of 2·5 points or less for IPSS and 4 mL per s or less for Qmax. Analysis was done according to the intention-to-treat principle. The trial is registered with the ISRCTN Registry, ISRCTN00788389.

**Findings:**

Between July 23, 2014, and Dec 30, 2016, 410 men were randomly assigned to ThuVARP or TURP, 205 per study group. TURP was superior for Qmax (mean 23·2 mL per s for TURP and 20·2 mL per s for ThuVARP; adjusted difference in means −3·12, 95% CI −5·79 to −0·45). Equivalence was shown for IPSS (mean 6·3 for TURP and 6·4 for ThuVARP; adjusted difference in means 0·28, −0·92 to 1·49). Mean hospital stay was 48 h in both study groups. 91 (45%) of 204 patients in the TURP group and 96 (47%) of 203 patients in the ThuVARP group had at least one complication.

**Interpretation:**

TURP and ThuVARP were equivalent for urinary symptom improvement (IPSS) 12-months post-surgery, and TURP was superior for Qmax. Anticipated laser benefits for ThuVARP of reduced hospital stay and complications were not observed.

**Funding:**

UK National Institute for Health Research Health Technology Assessment Programme.

## Introduction

Benign prostatic obstruction is a common condition resulting from prostate enlargement, and can cause lower urinary tract symptoms or urinary retention, with a substantial effect on men's quality of life.^1^ Surgery to relieve the obstruction is indicated after failure of medication to improve voiding and to prevent the complications associated with benign prostatic obstruction. Bothersome lower urinary tract symptoms secondary to benign prostatic obstruction affect around 3% of men aged 45–49 years in the UK, increasing to more than 30% of men aged 85 years and older.^2^ With an ageing population in the UK, the number of patients with benign prostatic obstruction is expected to grow, increasing the need for surgery.^2^

Around 25 000 prostate operations are done annually in the UK to relieve benign prostatic obstruction. Transurethral resection of the prostate (TURP) has been the gold standard of surgery for over 40 years, accounting for around 80% of operations. TURP is generally a successful procedure but is associated with small but significant mortality (0·3% within 30 days) and morbidity risks including transurethral resection syndrome (absorption of irrigating fluid causing confusion and collapse), haemorrhage during the operation, and subsequent urinary tract infections.^3^

Various alternative surgeries, including laser techniques, have been developed over the past 20 years. However, uptake has been relatively slow in many parts of the world, including the UK, due in part to a long learning curve or inferior clinical outcomes, despite the commonly accepted advantages of laser prostatectomy, including lower risk of perioperative complications, shorter catheterisation time, and reduced hospital stay.^3^

**Research in context**

**Evidence before this study**

Transurethral resection of the prostate (TURP) is the standard operation for men with benign prostatic obstruction. This procedure is generally considered to be successful; however, it is associated with small but clinically significant risks of morbidity and mortality. Thulium laser transurethral vaporesection of the prostate (ThuVARP) is a laser procedure that vapourises and resects the prostate using a technique similar to TURP. The 2013 European Association of Urology (EAU) guidelines for the treatment and follow-up of non-neurogenic male lower urinary tract symptoms, including benign prostatic obstruction, formed the evidence base for the UNBLOCS study. The guidelines were based on a literature search including all articles published in English in PubMed, MEDLINE, Web of Science, and Cochrane databases. Based on the one randomised controlled trial and one non-randomised prospective controlled trial that had been done with small and medium-sized prostates, the EAU guidelines stated that ThuVARP showed equivalent efficacy to TURP. Moreover, ThuVARP achieved shorter catheterisation and hospitalisation times, with a lower level of adverse events than TURP (intra-operative and postoperative bleeding; level of evidence 1b). The 2010 National Institute for Health and Care Excellence (NICE) Clinical Guideline recommended that laser vapourisation or vaporesection techniques should only be offered as part of a randomised trial comparing these techniques with TURP because of the limited evidence base.

**Added value of this study**

To our knowledge, the UNBLOCS trial is the largest randomised trial to compare ThuVARP to standard TURP. This masked trial included patients with urinary retention, who are frequently excluded from benign prostatic obstruction surgery trials, and made extensive use of patient-reported outcome measures. In contrast to previous studies, the trial showed that ThuVARP and TURP procedures achieve equivalent patient-reported urinary symptoms after surgery (International Prostate Symptom Score), but that TURP is superior in the urinary flow rate achieved (Qmax). TURP and ThuVARP had similar results across almost all other clinical operative outcomes, including bleeding and complication rates, length of stay in hospital, and patient-reported urinary symptoms, sexual symptoms, quality of life, and satisfaction after surgery. Patients with urinary retention had similarly positive outcomes after benign prostatic obstruction surgery as patients with lower urinary tract symptoms, contrary to the commonly held urological belief that this population has poorer outcomes.

**Implications of all the available evidence**

Overall, both ThuVARP and TURP can be recommended as clinically effective procedures for relieving benign prostatic obstruction; however, TURP achieved a superior urinary flow rate. The potential advantages of ThuVARP in reducing blood loss and shortening hospital stay were not observed in this study. Our results suggest that it is appropriate that new treatment alternatives continue to be compared with the current standard of TURP, as per the NICE guidelines. Our trial results can be used to update the literature and urology guidelines, allowing patients to be more informed at the point of consent on the risks and benefits of such procedures, especially with regard to side-effects.

Although recommended by the UK National Institute for Health and Care Excellence (NICE) for several years, holmium laser enucleation of the prostate has not proved generalisable, requiring extensive experience, learning of a unique skill, and the need to morcelate the prostate within the bladder to extract the enucleated tissue. Therefore, NICE guidelines suggest performing holmium laser enucleation of the prostate at a centre specialising in the technique or with mentorship arrangements in place.^4^ Greenlight laser therapy is also approved by NICE but only vaporises the prostate without generating tissue for histology, with insufficient evidence for use in high-risk patients.^5^

In this study, we evaluate a laser technique called thulium laser transurethral vaporesection of the prostate (ThuVARP). ThuVARP uses a thulium-yttrium aluminum garnet fibre to deliver light of 2000 nm wavelength to vaporise and resect the prostate.^6^ Unlike other laser technologies, ThuVARP uses a surgical technique similar to TURP—ie, visual resection of prostatic tissue using a working element and resecting in so-called chips, which is taught to all urologists during training. The similarity in technique to TURP allows a short learning curve for surgeons (previously shown in the UNBLOCS trial),^7^ giving ThuVARP the potential for widespread adoption into clinical practice.

At the time this trial was designed, ThuVARP showed positive outcomes in a randomised trial in China, with relatively small numbers and short follow-up, but without evaluation of all key outcomes.^8^ European Association of Urology guidelines concluded that ThuVARP showed equivalent efficacy compared with TURP, but patients had shorter catheterisation and hospitalisation times, with lower adverse events than for TURP (intraoperative and postoperative bleeding; level of evidence 1b).^9^ However, 2010 NICE guidelines recommended that laser vaporisation or vaporesection techniques should only be offered as part of a randomised trial comparing these techniques with TURP, because of the restricted evidence base.^4^ In the UNBLOCS trial, we chose ThuVARP for comparison against TURP in a pragmatic randomised trial because of the potential for improved clinical outcomes paired with ease of generalisability.

## Methods

### Study design and participants

The UNBLOCS study is a multicentre, pragmatic, randomised, parallel-group equivalence trial of ThuVARP versus standard TURP. We aimed to determine whether ThuVARP was equivalent to TURP in men with benign prostatic obstruction, in terms of the patient-reported International Prostate Symptom Score (IPSS) and maximum urine flow rate (Qmax). The trial was done in four university teaching hospitals and three district general hospitals in the UK. Men presenting in secondary care with either bothersome lower urinary tract symptoms or urinary retention, secondary to benign prostatic obstruction, and suitable for TURP surgery (having failed conservative and medical therapy), were recruited. Men were excluded if they had neurogenic lower urinary tract symptoms, prostate cancer, previous prostate or urethral surgery, a prostate specific antigen level outside the normal age-related range without prostate cancer excluded, or were unable to give informed consent or complete trial documentation.

Ethics approval was received from the NRES Committee South Central—Hampshire B Ethics Committee (reference 13/SC/0644). The Consolidated Standards of Reporting of Trials (CONSORT) guidelines for outcomes were followed. All patients provided written informed consent and the trial was conducted according to Good Clinical Practice guidelines.

The study protocol is available online and has been previously published.^10^

### Randomisation and masking

Patients were randomly assigned (1:1) to TURP or ThuVARP through an automated, computer-generated web or telephone randomisation system. Randomisation was done at the point of surgery by the surgeon or research nurse, was stratified by centre and presentation (bothersome lower urinary tract symptoms or urinary retention), and random blocking was used.

Patients remained masked to their allocation until completion of follow-up. Randomisation was done after the patient was anaesthetised to facilitate patient blinding and to conceal allocation until the patient was in surgery. Ward staff, theatre notes, and discharge letters were blinded to avoid inadvertent unmasking of patients after surgery, with additional measures to avoid unmasking during surgery in patients undergoing spinal anaesthetic. Participants were informed of the type of surgery received after completion of their 12-month follow-up. Patient masking was assessed by asking patients whether they knew their allocation in their 12-month questionnaire. Surgeons doing the interventions and outcome assessors were not masked.

### Procedures

Given the pragmatic design of the trial, centres used their usual TURP procedure (monopolar or bipolar).^10^ We used a LISA Revolix 120W thulium laser system (Katlenburg-Lindau, Germany) for ThuVARP using a 550 μm fibre.^6^ All trial surgeons underwent training on the ThuVARP technique for the study and the competence of each surgeon was assured by an independent assessor before entering patients into the trial.

Patient clinical outcomes were recorded at baseline (pre-surgery), pre-operatively, and peri-operatively during their hospital stay for their allocated surgery, and at 3 months and 12 months post-surgery. Qmax, post-void residual, and voided volume were measured before surgery. Post-void residual and voided volume were measured post-operatively, and Qmax, post-void residual, and voided volume were measured at 3 months and 12 months post-surgery. Blood parameters were also measured at baseline and postoperatively.

Patient-reported outcomes were collected by paper questionnaires completed by participants at the baseline clinic visit, by post at 6 weeks, and at their 3-month and 12-month clinic visits. Patients received a single reminder if questionnaires were not returned at 6 weeks and 3 months, and two reminders at 12 months. Baseline questionnaire data and urinary flow measures were collected for patients with lower urinary tract symptoms; however, equivalent data could not be collected for catheterised patients with urinary retention because of their inability to void.

Sites followed their usual practice for management of patients on anticoagulation medication. Prostate size was assessed by digital rectal examination.

### Outcomes

The co-primary outcomes were maximum urine flow rate (Qmax; mL per s) and IPSS score at 12 months postsurgery. IPSS is a well-established and validated patient-reported outcome, with a score that ranges from 0 to 35, with higher scores indicating more severe urinary symptoms.^11^ Qmax is a urodynamic clinical measure that is used across benign prostatic obstruction trials.

Surgical secondary outcomes comprised complications occurring after leaving recovery until completion of 12-month follow up (Clavien-Dindo classification^12^) and length of hospital stay. Perioperative complications were reported separately without Clavien-Dindo classification. The additional secondary outcomes of postoperative catheterisation time (time to successful trial without catheter and ongoing catheter use), urinary post-void residual, blood loss during surgery (change in haemoglobin and blood transfusion rate), and absorption of irrigation fluid (change in serum sodium) were added as a protocol amendment (approved June 1, 2017).

Lower urinary tract symptoms were measured using the IPSS and International Consultation on Incontinence Questionnaire Male Lower Urinary Tract Symptoms Module (ICIQ-MLUTS).^13^ Urinary symptoms included voiding symptoms (related to passing urine, such as hesitancy and poor urinary flow) and storage symptoms (related to urine bladder storage, such as frequency and nocturia). Sexual function was measured by the International Consultation on Incontinence Questionnaire Male Sexual Matters Associated with Lower Urinary Tract Symptoms Module (ICIQ-MLUTSsex)^14^ and the International Index of Erectile Function (IIEF).^15^ Quality of life was measured by the IPSS QoL subscore and the International Consultation on Incontinence Questionnaire Lower Urinary Tract Symptoms Quality of Life Module (ICIQ-LUTSqol),^16^ and patient satisfaction with surgery by the ICIQ Satisfaction questionnaire.^17^ All questionnaires were fully validated, with the exception of the partly validated ICIQ Satisfaction questionnaire. Patient-reported outcomes for participants with an indwelling catheter were not included in the analysis at baseline (185 of 406 patients) or 12 months after surgery (six of 387 patients). Resource use and qualitative interview secondary outcomes are reported elsewhere.^18^

### Statistical analysis

The sample size calculation assumed that men randomly assigned to ThuVARP should have clinical outcomes equivalent to those who were randomised to TURP. For primary outcomes, differences in IPSS score of no more than 2·5 points and no more than 4 ml per sec for Qmax were hypothesised as suggesting equivalence. These hypotheses were based on the minimally clinically important differences in the literature and discussions with urologists on clinically relevant cutoffs. Further details on these justifications can be found in the statistical analysis plan.^19^ Assuming SDs of 9 ml per sec for Qmax and 5 units for IPSS, the target sample size for patients needed to complete the 12-month follow-up was 163 per group. This sample size provided 85% power to show equivalence for Qmax and just over 90% power for IPSS, at a two-sided α of 5%. Assuming 20% loss to follow-up, we needed to recruit 410 men.

The main statistical analyses were prespecified using a statistical analysis plan.^19^ As the primary outcomes for this trial were testing for equivalence between the study groups, emphasis was placed on estimates and CIs and their distances from the prespecified equivalence margins. All analyses were done using intention-to-treat randomly allocated groups and, where possible, were adjusted for centre and patient diagnosis at baseline (lower urinary tract symptoms *vs* urinary retention).

Complications of treatment were also explored on an as-treated basis to identify any treatment-specific complications. Binary outcomes were presented as n (%) and continuous outcomes as mean (SD) or median (IQR), as appropriate. Baseline data were considered imbalanced by randomised group if there was more than 0·5 SD or an absolute difference of 10%.

In line with the protocol^10^ and statistical analysis plan,^19^ missing data were used in the comparison of IPSS scores and Qmax levels for the primary analysis of this trial. We used multiple imputation by chained equations to impute missing values for the primary outcomes and details can be found in the appendix (appendix p 2). The primary analyses of Qmax and IPSS were done with a linear regression model, adjusting for centre and baseline presentation (urinary retention *vs* lower urinary tract symptoms). Various sensitivity analyses were done and details of these can be found in the appendix (appendix p 2).

For all other secondary analyses, analyses were based on complete case analyses and assessing for superiority; therefore, estimates, CIs, and p values are presented. We explored complications using ordinal logistic regression to account for quantity and severity. Where patients had multiple complications, and therefore grading within one complication type, the highest was taken. Given the small number of complications, we did not adjust this analysis for centre or baseline diagnosis. We analysed additional clinical outcomes using linear, logistic, or ordinal logistic regression, as appropriate. We analysed time to successful trial without catheter using a Cox proportional hazards model. Where continuous outcomes were skewed, the median and IQR are presented. We used linear regression to allow adjustment; however, relevant model assumptions were checked along with comparisons to a non-parametric approach. Quintiles of post-void residual were calculated and analysed using ordinal logistic regression because of the high proportion of patients with zero post-void residual at 12 months.

Patient-reported outcomes were scored and analysed as recommended, including voiding and incontinence scores (ICIQ-MLUTS) and an overall erectile dysfunction score (IIEF). Dichotomous variables were also created to assist reporting^20^ (appendix p 5).

Each patient-reported outcome was compared between study groups at 12 months using linear and logistic regression as appropriate. Where the distributions of continuous variables were skewed, means (and SD) are presented to see beyond ceiling effects (eg, the median satisfaction score was 10 out of 10 for both groups). The adjusted p value from each regression model on skewed data was also compared with the one achieved from the Mann-Whitney test to ensure consistency. Where ordinal outcomes were dichotomised, to aid interpretation results were also compared on an ordinal scale to ensure consistency. Analyses were not adjusted for baseline measures of the patient-reported outcomes because of an inability to collect data from catheterised patients.

We used STATA version 15.1 for all analyses. The trial was overseen by an independent data monitoring committee and is registered with the ISRCTN Registry, ISRCTN00788389.

### Role of the funding source

The funder of the study had no role in study design, data collection, data analysis, data interpretation, or writing of the report. The corresponding author had full access to all the data in the study and had final responsibility for the decision to submit for publication.

## Results

**Figure 1:**
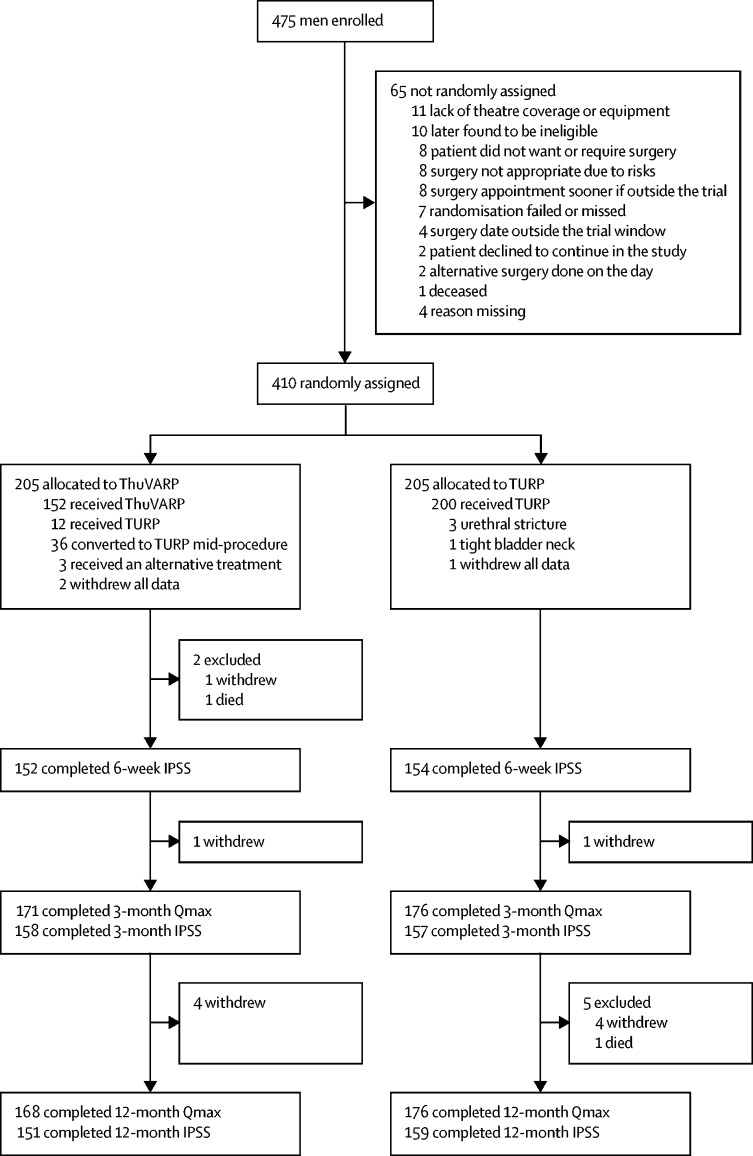
Trial profile ThuVARP=thulium laser transurethral vaporesection of the prostate. TURP=transurethral resection of the prostate. Qmax=maximum urinary flow rate. IPSS=International Prostate Symptom Score.

Between July 23, 2014, and Dec 30, 2016, we randomly assigned 410 men, 205 to each study group. Patient follow-up was completed in December, 2017. 152 (74%) of 205 participants allocated to ThuVARP and 200 (98%) of 205 participants allocated to TURP underwent their randomly assigned procedure (figure 1). Reasons for changes in treatment are listed in the appendix (p 5). Looking at conversions from ThuVARP to TURP mid-procedure, if the recruitment period was divided per surgeon into two halves, 13 (14%) of 94 procedures were converted in the first half and 22 (24%) of 90 procedures were converted in the second half. Overall, 16 participants withdrew from the study before their 12-month primary endpoint (three requested complete data withdrawal). At 12 months postsurgery, 310 (76%) of 410 participants completed the IPSS questionnaire and 344 (84%) had their Qmax recorded (figure 1).

Participant, clinical and patient-reported characteristics in the two trial groups were similar at baseline, with only painful ejaculation differing by more than an absolute difference of 10% (Table 1, Table 2). Around half the trial population presented with bothersome lower urinary tract symptoms and half with urinary retention, consistent with 213 (52%) of 406 patients being catheterised at baseline. Baseline urinary flow rates were generally poor, in line with the need for benign prostatic obstruction surgery, with a mean of around 9 mL per s. Patients also had a mean baseline IPSS score of 22, indicating severe symptoms.

**Table 1:** Baseline sociodemographic and clinical characteristics

			ThuVARP*(n=203)	TURP*(n=204)
Age, years			70·85 (7·85)	69·22 (7·91)
Bothersome lower urinary tract symptoms			94 (46%)	102 (50%)
Urinary retention			109 (54%)	102 (50%)
Ethnicity†				
	White		196 (98%)	197 (98%)
	Black, African, or Caribbean		1 (<1%)	1 (<1%)
	Asian or British Asian		2 (1%)	1 (<1%)
	Mixed or multiple ethnicities		0	1 (<1%)
	Other		0	1 (<1%)
BMI (on day of surgery)‡, kg/m^2^			28·54 (4·16)	27·83 (4·32)
Comorbidities at baseline (from the Charlson Comorbidity Index)				
	None		113 (56%)	115 (56%)
	One		57 (28%)	58 (28%)
	More than one		33 (16%)	31 (15%)
Catheterisation status (on the day of surgery)				
	No catheter§		96 (48%)	97 (48%)
	Catheterised		106 (52%)	107 (52%)
		Intermittent	4 (4%)	10 (9%)
		Indwelling	93 (88%)	92 (86%)
		Type not disclosed¶	9 (8%)	5 (5%)
Urinary measures				
	Maximum flow rate, Qmax‖		8·90 (5·90)	8·00 (6·00)
	Post-void residual, mL‖		157 (53–285)	140 (80–300)
	Voided volume, mL‖		186 (110–251)	181 (117–244)
	Prostate size, g**		35 (25–50)	40 (20–50)
	Patient has had urodynamics††		37 (19%)	44 (23%)

Data are mean (SD), n (%), or median (IQR). ThuVARP=thulium laser transurethral vaporesection of the prostate. TURP=transurethral resection of the prostate. BMI=body-mass index. Qmax=maximum urinary flow rate.

* Two patients in the ThuVARP group and one patient in the TURP group requested for all their data to be withdrawn; therefore, the patient totals are 203 and 204, respectively.

† Data are missing for five patients in the ThuVARP group and three patients in the TURP group.

‡ Data are missing for ten patients in the ThuVARP group and ten patients in the TURP group.

§ Data are missing for one patient in the ThuVARP group.

¶ Treated as indwelling for the imputed primary analysis, as these patients were unable to void at baseline.

‖ Urinary measures were excluded for men with indwelling catheters at baseline; the minimum numbers who completed baseline urinary measures were 92 and 99 for ThuVARP and TURP, respectively.

** Data were missing on prostate size for 17 patients in the ThuVARP group and 23 in the TURP group.

†† Data were missing on urodynamics for 11 patients in the ThuVARP group and 13 in the TURP group.

**Table 2:** Baseline patient-reported outcome measures of non-catheterised patients

		**Score range (low to high severity)**	**ThuVARP (n=100)**	**TURP (n=107)**
IPSS—urinary symptoms*				
IPSS storage subscale		0–15	9·51 (3·07)	10·09 (3·14)
	Frequency	0–5	3·63 (1·25)	3·83 (1·42)
	Urgency	0–5	2·97 (1·64)	3·30 (1·44)
	Nocturia	0–5	2·97 (1·34)	2·83 (1·35)
IPSS voiding subscale		0–20	12·17 (4·55)	12·45 (4·63)
	Incomplete emptying	0–5	3·12 (1·72)	3·29 (1·60)
	Intermittency	0–5	2·94 (1·61)	2·99 (1·58)
	Weak stream	0–5	3·91 (1·36)	3·80 (1·30)
	Straining	0–5	2·20 (1·77)	2·37 (1·79)
Total IPSS score		0–35	21·74 (6·37)	22·56 (6·78)
**ICIQ-MLUTS—urinary symptoms**†				
Voiding score		0–20	11·62 (4·35)	11·78 (3·92)
Incontinence score		0–24	5·75 (3·42)	6·10 (3·85)
Daytime frequency (>8 times)		NA	42 (52%)	56 (58%)
Nocturia (>1 time per night)		NA	75 (82%)	81 (84%)
**ICIQ MLUTS—sexual function**‡				
Reduced or no erections		NA	65 (76%)	65 (71%)
Reduced or no ejaculation		NA	73 (86%)	75 (84%)
Painful ejaculation		NA	13 (18%)	30 (35%)
Urinary symptoms affected sex life		NA	56 (68%)	62 (70%)
**International Index of Erectile Function**—**sexual function**§				
Total score		25–5	14·11 (6·51)	16·49 (6·17)
**IPSS—quality of life**¶				
IPSS quality-of-life score		0–6	4·89 (1·11)	5·01 (1·01)
**ICIQ-LUTS quality-of-life module—presence of limitations**‖				
Role limitations		NA	73 (83%)	79 (81%)
Physical limitations		NA	77 (85%)	84 (87%)
Social limitations		NA	57 (64%)	76 (80%)
Personal relationships		NA	63 (84%)	67 (81%)
Emotions		NA	68 (77%)	84 (89%)
Sleep or energy		NA	89 (99%)	91 (86%)
Severity measures		NA	78 (90%)	82 (86%)
ICIQ-LUTS quality of life—urinary symptom effect on**				
Getting embarrassed		NA	59 (66%)	66 (68%)
Overall interference with everyday life		0–10	6·02 (2·87)	6·49 (2·94)

Data are mean (SD) or n (%), unless otherwise indicated. Based on exclusion of total withdrawals and numbers with indwelling catheters, the maximum number of potential responders was 100 for ThuVARP and 107 for TURP. ThuVARP=thulium laser transurethral vaporesection of the prostate. TURP=transurethral resection of the prostate. IPSS=International Prostate Symptom Score. ICIQ-MLUTS=International Consultation on Incontinence Questionnaire Male Lower Urinary Tract Symptoms Module. ICIQ-LUTS=International Consultation on Incontinence Questionnaire Lower Urinary Tract Symptoms. NA=not applicable.

* Minimum numbers analysed were 86 patients for ThuVARP and 89 patients for TURP, with larger scores indicating more severe symptoms.

† Minimum numbers analysed were 89 patients for ThuVARP and 96 patients for TURP, with larger scores indicating more severe symptoms.

‡ Minimum numbers analysed were 72 patients for ThuVARP and 85 patients for TURP.

§ Numbers analysed were 65 patients for ThuVarp and 74 patients for TURP; lower scores indicate more severe erectile dysfunction (5–7=severe, 8–11=moderate, 12–16=mild to moderate, 17–21=mild, and 22–25=none).

¶ Numbers analysed were 90 patients for ThuVARP and 97 patients for TURP; higher scores indicate poorer quality of life.

‖ Minimum numbers analysed were 75 patients for ThuVARP and 83 patients for TURP.

** Minimum numbers analysed were 90 patients for ThuVARP and 95 patients for TURP; higher scores indicate poorer quality of life.

**Figure 2:**
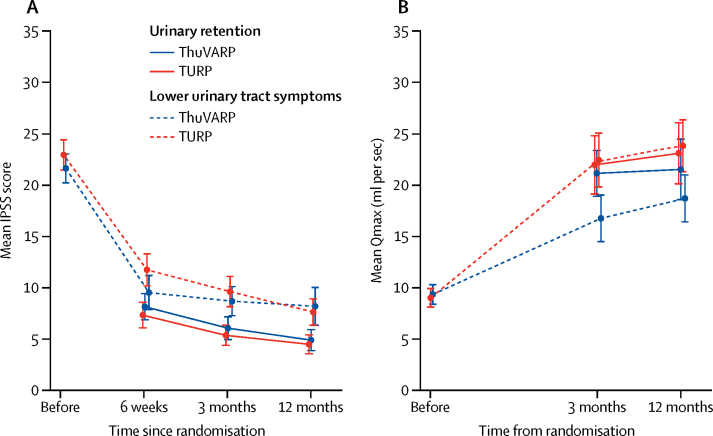
IPSS and Qmax over time, across baseline diagnosis and randomisation group (A) Total IPSS score. (B) Qmax level in mL per s. This figure is based on complete cases only, with no imputation for missing values, and patients with urinary retention who completed baseline scores have been removed from the analysis (n=13 for IPSS and n=21 for Qmax). IPSS=International Prostate Symptom Score. ThuVARP=thulium laser transurethral vaporesection of the prostate. TURP=transurethral resection of the prostate. Qmax=maximum urinary flow rate.

The two procedures were equivalent (margin 2·5) for IPSS at 12 months post-surgery, with an adjusted difference in means of 0·28 points (95% CI −0·92 to 1·49; table 3). ThuVARP had a lower mean Qmax at 12 months compared with TURP (adjusted difference in means of −3·12, 95% CI −5·79 to −0·45), with the lower CI outside the equivalence range (–4 to 4), indicating the treatments are non-equivalent. Changing to superiority testing (without statistical penalty after an equivalence analysis),^21^ suggested that TURP was superior to ThuVARP for Qmax (table 3). Per-protocol and complier average causal effect models strengthened the results of the main intention-to-treat analysis (appendix p 6). For Qmax, the results from the per-protocol and complier average causal effect model analyses suggested an even greater advantage to TURP (appendix p 6). All other prespecified sensitivity analyses agreed with the results of the primary analysis. We also saw no evidence that any prespecified subgroups altered these results (eg, age, lower urinary tract symptoms *vs* urinary retention, comorbidities, or prostate size; appendix p 7). While IPSS and Qmax levels could not be achieved at baseline for those diagnosed with urinary retention, the benefits of TURP were more apparent for participants with lower urinary tract symptoms (figure 2; appendix p 8). However, subgroup interaction tests at 12 months, although underpowered, could not consolidate this potential difference (p=0·888 for IPSS; p=0·189 for Qmax).

**Table 3:** 12-month results for primary endpoints after randomisation to ThuVARP or TURP

	**n (ThuVARP:TURP)**	**ThuVARP, mean (SD)**	**TURP, mean (SD)**	**Crude difference in means (95% CI)**	Adjusted difference in means*(95% CI)
IPSS score	197:199	6·43 (6·79)	6·26 (5·79)	0·16 (−1·08 to 1·41)	0·28 (−0·92 to 1·49)
Qmax	197:199	20·16 (16·88)	23·24 (13·28)	−3·08 (−5·75 to −0·41)	−3·12 (−5·79 to −0·45)

ThuVARP=thulium laser transurethral vaporesection of the prostate. TURP=transurethral resection of the prostate. IPSS=International Prostate Symptom Score. Qmax=maximum urinary flow rate.

* Adjusted for centre and baseline diagnosis.

There were similar levels of perioperative and post-operative complications between the study groups (96 [47%] of 203 men in the TURP group *vs* 91 [45%] of 204 men in the ThuVARP group had at least one post-operative complication), no cases of transurethral resection syndrome in either group, and no evidence of a difference in bleeding (Table 4, Table 5; appendix p 8). Mean hospital stay was 48 h for both groups and catheter requirement rates were low and similar between procedures. Although the proportion of men with 0 post-void residual was almost equal in both groups (39 [23%] of 169 participants in the ThuVARP group versus 39 [22%] of 176 participants in the TURP group), we observed evidence to suggest that post-void residual levels were higher in the ThuVARP group (table 5). Two men received a repeat TURP procedure for benign prostatic obstruction in the TURP group compared with three men in the ThuVARP group by 12-months post-surgery (of those who received their original benign prostatic obstruction surgery). The median energy delivered for ThuVARP was 148 kJ (IQR 120–197; range of 20–987) based on 101 (66%) of 152 ThuVARP patients.

**Table 4:** Postoperative surgical complications (Clavien-Dindo scores per patient)

	**ThuVARP**	**TURP**	Odds ratio (95% CI)*	**p value**
**Bleeding requiring haemoglobin measure**				
Not experienced	188 (94%)	189 (95%)	1·00 (0·42–2·35)	0·992
Grade I	10 (5%)	8 (4%)	..	..
Grade II	0	2 (1%)	..	..
Grade IIIb	1 (1%)	1 (1%)	..	..
**Transurethral resection syndrome**†				
Not experienced	203 (100%)	203 (100%)	..	..
**Catheter misplacement**				
Not experienced	198 (100%)	199 (>99%)	..	..
Grade II	0	1 (1%)	..	..
**Clot retention**				
Not experienced	190 (95%)	189 (94%)	0·74 (0·30–1·79)	0·498
Grade I	9 (5%)	9 (4%)	..	..
Grade II	0	2 (1%)	..	..
Grade IIIb	0	1 (<1%)	..	..
**Urethral stricture**				
Not experienced	191 (96%)	195 (98%)	1·43 (0·45–4·59)	0·546
Grade I	0	2 (1%)	..	..
Grade II	0	0	..	..
Grade IIIa	4 (2%)	0	..	..
Grade IIIb	3 (2%)	3 (2%)	..	..
**Urinary tract infection**				
Not experienced	131 (68%)	136 (68%)	1·02 (0·67–1·55)	0·938
Grade I	10 (5%)	11 (6%)	..	..
Grade II	51 (26%)	53 (27%)	..	..
Grade IVb	1 (1%)	0	..	..
**Pyrexia of unknown region**				
Not experienced	188 (97%)	190 (98%)	1·50 (0·42–5·41)	0·533
Grade I	2 (1%)	0	..	..
Grade II	4 (2%)	4 (2%)	..	..
**Sepsis, septicaemia, or abscess**				
Not experienced	190 (99%)	189 (98%)	0·50 (0·09–2·76)	0·427
Grade II	1 (1%)	3 (2%)	..	..
Grade Iva	0	1 (1%)	..	..
Grade IVb	1 (1%)	0	..	..
**Other infection**				
Not experienced	186 (97%)	187 (96%)	0·71 (0·22–2·29)	0·570
Grade I	1 (1%)	0	..	..
Grade II	4 (2%)	7 (4%)	..	..

Data are n (%), unless otherwise indicated. Data exclude perioperative complications. When patients had multiple events or grades within one complication type, the highest was taken. For each item, patients were not included if data were missing on complications in either their postoperative, 3-month, or 12-month clinical report form. Categories of Clavien-Dindo grading are missing when they were not experienced by at least one patient. ThuVARP=thulium laser transurethral vaporesection of the prostate. TURP=transurethral resection of the prostate.

* Ordinal logistic regression not adjusted for centre or baseline diagnosis given the small number of events.

† Transurethral resection syndrome data were not collected at 3 months or 12 months.

**Table 5:** Secondary surgical outcomes

		**n (ThuVARP:TURP)**	**ThuVARP**	**TURP**	Adjusted comparison*(95% CI)	**p value**
Total number of complications during the 12-month period†		..	..	..	1·10 (0·75 to 1·63)	0·623
	0	NA	107 (53%)	113 (55%)	..	..
	1	NA	28 (14%)	27 (13%)	..	..
	>1	NA	68 (34%)	64 (31%)	..	..
Surgery outcomes						
	Length of hospital stay, h‡	198:198	48 (29 to 58)	48 (29 to 61)	−3·28 (−9·61 to 3·06)	0·310
	Transfusion required, yes *vs* no§	200:202	3 (2%)	4 (2%)	0·79 (0·17 to 3·62)	0·765
	Postoperative catheter time, days¶	195:198	2 (1 to 5)	2 (1 to 4)	1·02 (0·83 to 1·26)	0·830
	Catheter required at 3 months§	196:201	5 (3%)	5 (2%)	0·99 (0·28 to 3·49)	0·988
	Catheter required at 12 months§	192:195	4 (2%)	2 (1%)	1·95 (0·35 to 10·82)	0·446
	Haemoglobin—blood loss, g/L‡‖	146:138	−6 (−13 to −1)	−8 (−16 to −2)	0·88 (−2·14 to 3·89)	0·568
	Serum sodium, mmol/L‡‖	141:138	−2 (−4 to −1)	−3 (−4 to −1)	0·40 (−0·34 to 1·14)	0·290
Post-void residual at 12 months, mL**		..	..	..	1·46 (1·00 to 2·15)	0·053
	Quintile 1 (range 0 to 0)	NA	39 (23%)	39 (22%)	..	..
	Quintile 2 (range 2 to 34)	NA	21 (12%)	39 (22%)	..	..
	Quintile 3 (range 35 to 71)	NA	35 (21%)	35 (20%)	..	..
	Quintile 4 (range 72 to 140)	NA	32 (19%)	36 (20%)	..	..
	Quintile 5 (range 141 to 1000)	NA	42 (25%)	27 (15%)	..	..

Data are n (%) or median (IQR), unless otherwise indicated. ThuVARP=thulium laser transurethral vaporesection of the prostate. TURP=transurethral resection of the prostate. NA=not applicable.

* Adjustment for centre and baseline diagnosis.

† Ordinal logistic regression comparing 0, 1, and >1 complications; to ensure all complications were captured, patients were included if they had at least one non-missing complication.

‡ Linear regression for continuous outcomes.

§ Logistic regression for binary outcomes, not adjusted for centre because of perfect prediction (catheter outcomes refer to indwelling catheters only).

¶ Analysed using a Cox proportional hazards model.

‖ Negative values indicate that levels collected postoperatively were lower than those collected at baseline.

** Analysed using ordinal logistic regression.

Questionnaire response rates at 12-months postsurgery varied across measures and were between 53% (IIEF; 218 participants) and 83% (ICIQ satisfaction; 340 participants), with over 75% of patients reporting on their urinary symptoms. Urinary symptoms were generally similar between the two study groups, with improvement from baseline apparent in patients with lower urinary tract symptoms (table 6; appendix pp 3, 9). However, TURP appeared to be slightly more effective in reducing the proportion of men reporting nocturia at 12 months, with 72 (44%) men getting up to urinate more than once per night in the ThuVARP group compared with 63 (37%) in the TURP group. In an ordinal scale analysis (0–5 times per night) the p value for nocturia was 0·031.

**Table 6:** Urinary symptoms and sexual function in all trial patients (presenting with lower urinary tract symptoms or urinary retention) at 12 months after surgery

		**Score range (low to high severity)**	**ThuVARP (n=190)**	**TURP (n=195)**	Adjusted difference*(95% CI)	p value*
**IPSS—urinary symptoms**†						
IPSS storage subscale		0–15	3·82 (3·16)	3·54 (2·76)	0·36 (−0·25 to 0·98)	0·245
	Frequency	0–5	1·28 (1·33)	1·25 (1·28)	..	..
	Urgency	0–5	0·90 (1·38)	0·84 (1·20)	..	..
	Nocturia	0–5	1·62 (1·13)	1·43 (1·10)	..	..
IPSS voiding subscale		0–20	2·58 (3·78)	2·51 (3·40)	0·13 (−0·63 to 0·88)	0·740
	Incomplete emptying	0–5	0·78 (1·16)	0·76 (1·03)	..	..
	Intermittency	0–5	0·63 (1·18)	0·68 (1·19)	..	..
	Weak stream	0–5	0·85 (1·36)	0·73 (1·16)	..	..
	Straining	0–5	0·33 (0·84)	0·42 (0·93)	..	..
IPSS total score		0–35	6·29 (6·22)	6·03 (5·21)	0·43 (−0·78 to 1·64)	0·484
**ICIQ-MLUTS—urinary symptoms**‡						
Voiding score		0–20	3·14 (3·40)	3·00 (3·07)	0·15 (−0·53 to 0·82)	0·666
Incontinence score		0–24	2·40 (2·65)	2·23 (2·37)	0·22 (−0·30 to 0·75)	0·406
Daytime frequency (>8 times)		NA	20 (12%)	18 (10%)	1·20 (0·61 to 2·39)	0·597
Nocturia (>1 time per night)		NA	72 (44%)	63 (37%)	1·47 (0·93 to 2·34)	0·102
**ICIQ-MLUTS—sexual function**§						
Reduced or no erections		NA	101 (70%)	113 (74%)	0·79 (0·47 to 1·31)	0·356
Reduced or no ejaculation		NA	129 (93%)	136 (92%)	1·13 (0·47 to 2·71)	0·780
Painful ejaculation		NA	8 (7%)	17 (12%)	0·55 (0·22 to 1·32)	0·179
Urinary symptoms affected sex life		NA	74 (56%)	88 (61%)	0·81 (0·50 to 1·31)	0·399
**International Index of Erectile Function—sexual function**¶						
Total score		25–5	14·18 (7·46)	15·14 (7·34)	−0·95 (−2·95 to 1·05)	0·348

Data are n (%) or mean (SD), unless otherwise indicated. Based on exclusion of total withdrawals and numbers with indwelling catheters, the maximum number of potential responders was 190 for ThuVARP and 195 for TURP. ThuVARP=thulium laser transurethral vaporesection of the prostate. TURP=transurethral resection of the prostate. IPSS=International Prostate Symptom Score. ICIQ-MLUTS=International Consultation on Incontinence Questionnaire Male Lower Urinary Tract Symptoms Module. NA=not applicable.

* ThuVARP compared with TURP, adjusted for centre and baseline diagnosis, using linear, logistic, or ordinal logistic regression; where data were skewed Mann-Whitney tests were used to ensure that conclusions drawn were appropriate.

† Minimum numbers analysed were 151 patients for ThuVARP and 159 patients for TURP, with larger scores indicating more severe symptoms.

‡ Minimum numbers analysed were 164 patients for ThuVARP and 172 patients for TURP, with larger scores indicating more severe symptoms.

§ Binary sexual patient reported outcomes were not adjusted for centre because of perfect prediction; minimum numbers analysed were 118 patients for ThuVARP and 139 patients for TURP.

¶ Numbers analysed were 100 patients for ThuVARP and 118 patients for TURP; lower scores indicate more severe erectile dysfunction (5–7=severe, 8–11=moderate, 12–16=mild to moderate, 17–21=mild, and 22–25=none).

Sexual dysfunction after surgery was very prevalent and generally similar between the two surgical groups at 12 months. A marginally lower level of painful ejaculation was observed in the ThuVARP group compared with the TURP group after surgery, but a difference between the groups was also apparent at baseline (Table 2, Table 6). At baseline, sexual dysfunction symptoms were common, with a high mean IIEF score, and 130 (70%) of 177 men had reduced or no erections. Overall there was little change in sexual symptoms after surgery in patients with lower urinary tract symptoms (appendix pp 4, 10). When comparing IIEF scores at baseline and 12-months post-surgery in a post-hoc analysis (appendix p 10), 6 (24%) of 25 patients with lower urinary tract symptoms without sexual dysfunction reported at baseline had developed between mild and moderate symptoms after surgery. 22 (31%) of 70 patients with sexual dysfunction at baseline had increased sexual dysfunction after surgery. By contrast, 16 (23%) patients with sexual dysfunction at baseline had improved sexual symptoms after surgery, with 32 (46%) of 70 patients' symptoms remaining unchanged.

In general, there was no difference in quality of life at 12-months postsurgery between the two study groups (table 7). 277 (82%) of 339 patients who answered the IPSS quality-of-life question at 12 months said they would be “mostly satisfied”, ”pleased”, or ”delighted” if they were to spend the rest of their lives with their urinary condition the way it is. Quality of life improved from baseline in patients with lower urinary tract symptoms (appendix p 3). Men in both study groups were satisfied with their treatment, with a mean satisfaction score of over 8·5 of 10, and 315 (93%) of 339 reported that they would definitely have the procedure again if required (table 7).

**Table 7:** Quality of life and satisfaction in all trial patients (presenting with lower urinary tract symptoms or urinary retention) 12 months after surgery

		**ThuVARP (n=190)**	**TURP (n=195)**	Adjusted difference*(95% CI)	p value*
**IPSS—quality of life**†					
IPSS quality of life		1·22 (1·67)	1·08 (1·46)	0·17 (−0·15 to 0·49)	0·294
**ICIQ-LUTSqol—presence of limitations**‡					
Role limitations		40 (24%)	39 (23%)	1·11 (0·66 to 1·87)	0·690
Physical limitations		59 (36%)	55 (32%)	1·24 (0·77 to 2·00)	0·374
Social limitations		30 (18%)	33 (19%)	0·97 (0·56 to 1·69)	0·911
Personal relationships		76 (66%)	81 (63%)	1·18 (0·69 to 2·02)	0·555
Emotions		42 (26%)	52 (30%)	0·86 (0·52 to 1·42)	0·552
Sleep or energy		116 (72%)	129 (74%)	0·91 (0·55 to 1·50)	0·710
Severity measures		76 (47%)	97 (58%)	0·65 (0·41 to 1·03)	0·067
**ICIQ-LUTSqol—urinary symptom effect on**§					
Getting embarrassed		23 (14%)	37 (21%)	0·61 (0·34 to 1·11)	0·108
Overall interference with everyday life		1·33 (2·39)	1·42 (2·27)	−0·07 (−0·55 to 0·41)	0·778
**ICIQ satisfaction**¶					
Overall, how satisfied were you with the treatment or procedure?		..	..	−0·21 (−0·65 to 0·22)	0·338
	On a scale of 0 (not) to 10 (very)	8·67 (2·42)	8·88 (1·92)	..	..
If you were in the same situation again would you still have the same treatment or procedure?		..	..	1·90 (0·78 to 4·59)	0·156
	Yes, definitely or probably	150 (91%)	165 (95%)	..	..
	Not sure	11 (7%)	5 (3%)	..	..
	No, definitely or probably not	4 (2%)	4 (2%)	..	..

Data are mean (SD) or n (%), unless otherwise indicated. Based on exclusion of total withdrawals and numbers with indwelling catheters, the maximum number of potential responders was 190 for ThuVARP and 195 for TURP. ThuVARP=thulium laser transurethral vaporesection of the prostate. TURP=transurethral resection of the prostate. IPSS=International Prostate Symptom Score. ICIQ-LUTSqol=International Consultation on Incontinence Questionnaire Lower Urinary Tract Symptoms Quality of Life Module.

* ThuVARP compared with TURP, adjusted for centre and baseline diagnosis, using linear, logistic, or ordinal logistic regression.

† Numbers analysed were 164 patients for ThuVARP and 175 patients for TURP; higher scores indicate poorer quality of life.

‡ Minimum numbers analysed were 115 patients for ThuVARP and 128 patients for TURP.

§ Minimum numbers analysed were 162 patients for ThuVARP and 173 for TURP; higher scores indicate poorer quality of life.

¶ Numbers analysed were 163 patients for ThuVARP and 177 for TURP.

238 (70%) of 342 patients reported not knowing which operation they received. When asked “How did you find out which type of surgery you had?”, 34 (10%) of 342 men said they had found out accidentally during clinic or general practitioner (GP) visit or had asked the GP, consultant, or nurse.

Although exploratory in nature, routine histology review from prostate tissue removed during surgery revealed that a higher number of men were diagnosed with prostate cancer in the TURP group than in the ThuVARP group (appendix p 11). 25 (13%) of 193 men were diagnosed with prostate cancer in the TURP group compared with 10 (5%) of 193 in the ThuVARP group (odds ratio 0·35, 95% CI 0·16–0·75), probably because of the prostate weight available after resection, with the median resected weight 65% smaller after ThuVARP than after TURP (7 g compared with 20 g).

## Discussion

In this study, ThuVARP was shown to be equivalent to TURP for patient-reported IPSS, but TURP was superior to ThuVARP for Qmax. However, the Qmax achieved by both procedures is considered clinically successful. Meta-analyses have identified several randomised trials^22^, ^23^, ^24^ comparing ThuVARP with TURP, although these were mostly done in Asia and on a smaller scale than this trial, and only one trial included patient blinding to an unspecified degree. In contrast to the results of this study, in one meta-analysis^23^ ThuVARP was significantly better in terms of both Qmax and IPSS, and ThuVARP and TURP were similar in the other two.^22^, ^24^

We observed no difference between TURP and ThuVARP in terms of length of hospital stay, blood transfusion rate, and drop in serum sodium after surgery in the UNBLOCS trial, unlike published meta-analyses in which ThuVARP was superior to TURP. The difference in hospital stay between previous studies and ours could be related to the previous studies being done in China, where most patients do not leave hospital until they can return to normal activities, thus increasing length of stay.^8^ In the UK, patients are encouraged to go home as soon as clinically appropriate, and patients are taught how to manage their catheters at home, until removal.

Overall, patient-reported outcomes were similar for TURP and ThuVARP for urinary and sexual symptoms, quality of life, and patient satisfaction, with no significant difference found between the study groups. However, there was a difference in nocturia between the groups, with an increased incidence at 12 months after surgery in the ThuVARP group, which warrants further investigation.

The effect of urinary symptoms on men's sex life before surgery is substantial, with little improvement after surgery in patients with lower urinary tract symptoms overall. The occurrence of new onset erectile dysfunction is commonly reported as between 2% and 10% when patients are informed of surgical risks.^25^ When comparing IIEF scores by severity at baseline, surgery results in variable outcomes for individual patients, including some patients having improvements in their sexual function. The high level of sexual symptoms in patients at baseline supports the routine measurement of baseline sexual function in benign prostatic obstruction trials, and the variable outcome after surgery should be included by clinicians in patient discussions.

The exploratory finding that the treatments differed in pathology diagnostic detection of prostate cancer might have been due to the reduced amount of tissue for histology from ThuVARP due to tissue vaporisation. The clinical significance of this finding is that a prostate cancer diagnosis might be missed because of the restricted histology available, although TURP is not part of the diagnostic pathway for prostate cancer.

A clinically important strength of UNBLOCS is the inclusion of patients with urinary retention (catheterised), who are usually excluded from benign prostatic obstruction surgical trials. To our knowledge, our study is unique in including patients with urinary retention and not restricting inclusion on prostate size. Therefore, published data on surgical outcomes for patients with urinary retention are scarce, with no comparative studies evaluating ThuVARP identified in a meta-analysis, and have been highlighted as a gap in the evidence base.^26^ However, our results show that patients with urinary retention do as well with de-obstructing surgery as men with lower urinary tract symptoms, contrary to the belief that such surgery might not improve symptoms, possibly because of a higher prevalence of detrusor underactivity or acontractile bladder in men with urinary retention. As detrusor contractility cannot be measured in men with urinary retention, it is reassuring that the catheter-free rate was 98% at 3-months and 12-months postsurgery. This trial highlights the importance of including patients with urinary retention in all trials of new benign prostatic obstruction surgical techniques, as they comprise 50% of the patient population.

Another strength of our study was participant masking, with only 10% of patients reporting active unmasking, minimising bias. Masking is often considered unfeasible for surgical trials;^22^ however, it is particularly important with a patient-reported co-primary outcome (IPSS). Additionally, as all surgeons could do both ThuVARP and TURP, surgeons were also masked to the randomised allocation until the time of surgery. This strategy reduced bias in subjective assessment of symptoms, and we would recommend randomisation at the point of patient anaesthesia for masking in future surgical trials, where logistically possible.

Further strengths of this study were the large sample size compared with previous trials, the European setting, and successful recruitment despite the logistics of randomisation at the point of surgery. The number of patients who withdrew was low and follow-up was high for a surgical trial with a patient-reported primary outcome.

The breadth of patient-reported outcomes has also produced novel findings, including data on incontinence in the ICIQ-MLUTS, which is absent from IPSS. Inclusion of the more comprehensive ICIQ-MLUTSsex adds value to the generalisability of the IIEF and allows patients to express the effect of their urinary symptoms on sexual function without the assumption of an active sex life. As only 218 (53%) of 410 patients responded for IIEF, data should be treated with caution because of a potential risk of bias. However, this trial presents a comprehensive account of sexual function before and after benign prostatic obstruction surgery, identified as being poorly reported in trials of emerging procedures, in which prevalence rather than incidence data are reported because of a lack of preoperative data.^27^, ^28^ Inclusion of the ICIQ-Satisfaction questionnaire also provided insight into the patient experience of, and satisfaction with, surgery.

Limitations of the trial were the inevitable inability to collect some baseline data from the catheterised population with urinary retention, preventing adjustment for baseline in the analysis. However, this limitation is outweighed by increased generalisability of the results. Before surgery, prostate volume was estimated by digital rectal examination rather than measured using transrectal ultrasound and prostate-specific antigen testing and invasive urodynamics were not routinely done; however, this strategy mimics routine pragmatic clinical practice in the UK. The small number of men of non-white race included in the study is also a limitation.

The rate of conversion from ThuVARP to TURP during the trial could also be considered a limitation; however, this pragmatic trial reflects the real-life scenario should this laser technique be introduced into clinical practice. Additionally, per protocol and complier average causal effect sensitivity analyses accounted for crossover and had similar results to the main analysis. The size of the prostate also resulted in nine conversions to TURP, which might reflect the lack of trial exclusion criteria for patients with large prostates. Future research into the comparative effectiveness of ThuVARP and TURP in large prostates would be useful.

A further potential limitation was the differential previous experience of the surgeons of TURP and ThuVARP, with trial surgeons having done over 100 TURP procedures, but only between five and 12 ThuVARP procedures.^7^ However, all surgeons were independently assessed before undertaking trial laser procedures and we have shown that ThuVARP has a short learning curve,^7^ with surgical skills similar to TURP. In comparing the conversion rates of ThuVARP to TURP during the trial, there was no evidence of a learning curve effect, with the rate of conversions actually increasing as surgeons conducted more cases.

In conclusion, both ThuVARP and TURP can be recommended as clinically effective procedures for relieving benign prostatic obstruction, however TURP achieved a superior maximum urinary flow rate (Qmax). The potential advantages of ThuVARP in reducing blood loss and shortening hospital stay were not observed in this study. Our results suggest that it is appropriate that new treatment alternatives continue to be compared with the current standard of TURP, as per NICE guidelines. Our trial results could be used to update the literature and urology guidelines, allowing patients to be more informed at the point of consent on the risks and benefits of such procedures, especially with regard to side-effects.

## Data sharing

All data requests should be submitted to the corresponding author for consideration. Access to anonymised data might be granted following review.
