# Supplementary material for: Thulium laser transurethral vaporesection of the prostate versus transurethral resection of the prostate for men with lower urinary tract symptoms or urinary retention (UNBLOCS): a randomised controlled trial
Source: Lancet. 2020 Jul 4;396(10243):50–61. doi: 10.1016/S0140-6736(20)30537-7 (PMC7339133; doi:10.1016/S0140-6736(20)30537-7)
Supplement: Supplementary appendix [file mmc1.pdf]

THE LANCET

Supplementary appendix

This appendix formed part of the original submission and has been peer reviewed. We post it as supplied by the authors.

Supplement to: Hashim H, Worthington J, Abrams P, et al. Thulium laser transurethral vaporesection of the prostate versus transurethral resection of the prostate for men with lower urinary tract symptoms or urinary retention (UNBLOCS): a randomised controlled trial. *Lancet* 2020; **396:** 50–61.

Supplementary Material

UNBLOCS Trial Investigators

Chief Investigator: Hashim Hashim
Principal Investigators: Rupert Beck, Christopher Blake, Kim Davenport, Hashim Hashim, Oliver Kayes, Tobias Page, Jonathan Sullivan, Satchi Swami
Trial Manager: Jo Worthington
Trial Research Associate: Hilary Taylor
Senior Trial Manager/BRTC Co-Director: Athene Lane
Trial Statisticians: Sara Brookes, Chris Metcalfe, Grace Young
Urologists: Nicholas Cohen, Mathialagan Murugesan, Anthony Timoney
Research Nurse lead: Lyndsey Johnson
Site Research Nurses: Benita Adams, Angela Allan, Carol Brain, Fiona Hammonds, Joan Henderson, Paula Hilltout, Bernadette Kilbane, Leigh Morrison, Wendy Robson, Lorraine Wiseman, Vivian Zinyemba
Qualitative researchers: Nikki Cotterill, Rafiyah Khan, Alan Uren
Health Economists: Aideen Ahern, Aida Moure Fernandez, Sian Noble
Administrative support: Tom Steuart-Feilding, Christopher Pawsey, Julie Plant, Barbara Warnes
Data management: Mai Baquedano, David Carmichael

Trial sites and principal investigators

The NHS Trusts participating as sites in the trial were:

Gloucestershire Hospitals NHS Foundation Trust (PI, Mr Jonathan Sullivan/Miss Kim Davenport)
Great Western Hospitals NHS Foundation Trust (PI, Mr Rupert Beck)
North Bristol NHS Trust (PI, Prof Hashim Hashim)
NHS Grampian (PI, Mr Satchi Swami)
Royal Cornwall Hospitals NHS Trust (PI, Mr Christopher Blake)
The Leeds Teaching Hospital NHS Trust (PI, Mr Oliver Kayes)
The Newcastle upon Tyne Hospitals NHS Foundation Trust (PI, Mr Tobias Page)

Study oversight committees

We thank all the members of the study oversight committees for their valued contributions.

UNBLOCS Trial Steering Committee: Professor Tom McNicholas (Chair), Mr Malcolm Lucas, Dr Catrin Tudur-Smith, Dr Gordon Taylor and Dr Glyn Hayes

Data Monitoring and Ethics Committee: Dr Jonathan Cook, Mr Mohammed Belal and Mr Mark Stott.

Trial Management Group: Professor Hashim Hashim, Dr Athene Lane, Dr Sara Brookes, Professor Chris Metcalfe, Dr Sian Noble, Mr Toby Page, Professor Paul Abrams, Dr Nikki Cotterill, Dr Jo Worthington, Lyndsey Johnson, Hilary Taylor

Supplementary Methods

Multiple imputation

Multiple imputation by chained equations was used to impute missing values for the primary outcomes. Although the intention was to use all available time points to impute IPSS data, the model failed to converge due to the high collinearity between the measures at 6 weeks and 3 months. Instead, the model included baseline and 12 month follow-up variables to inform imputation. Trial arm, baseline diagnosis of LUTS or UR, baseline comorbidities, and age were included as complete variables. Where missing, indwelling catheter status (Y/N) was imputed at baseline and 12 months. Qmax and IPSS individual items were imputed at time points where patients did not have an indwelling catheter; using predictive mean matching and conditional imputation. Forty individual imputations were created and combined using Rubin's rules, using a pre-specified randomisation seed.

Sensitivity analyses

In total there were nine sensitivity analyses that were carried out on the primary outcomes, to test the robustness of the results, of which seven were pre-specified. A complete case analysis was carried out which utilized IPSS and Qmax data that was completed, with no imputation for missing data. A per protocol analysis only included those who received the treatment they were assigned to and a complier average causal effect which incorporated the randomised allocation as the instrumental variable and treatment received as the independent variable. Any patients who became unblinded before their 12 month period were removed in a sensitivity analysis.

Adjustments were made for baseline imbalance; differences of >0.5 standard deviations for continuous outcomes and 10% for categorical outcomes. As the primary analysis was unable to adjust for baseline, as patients with indwelling catheters could not provide an IPSS score or Qmax, a suitable baseline value was calculated. For Qmax, all patients with an indwelling catheter were given a value of zero. For IPSS, baseline scores were categorized for all men into mild (0-7), moderate (8-19) or severe (20-35). Men with indwelling catheters were placed in the severe category. A mixed-effects model, including surgeon as a random effect and centre as a fixed effect, was conducted to account for unobserved heterogeneity between surgeons.

Two post-hoc analyses were included to account for the unexpected skewness in the IPSS and Qmax data. Bootstrap regression was performed (4999 replications) as well as regression using log transformed values.

Supplementary Figures

Supplementary Figure 1 (S1). Urinary symptom scores over time for patients presenting with LUTS or UR at baseline (IPSS and ICIQ-MLUTS)

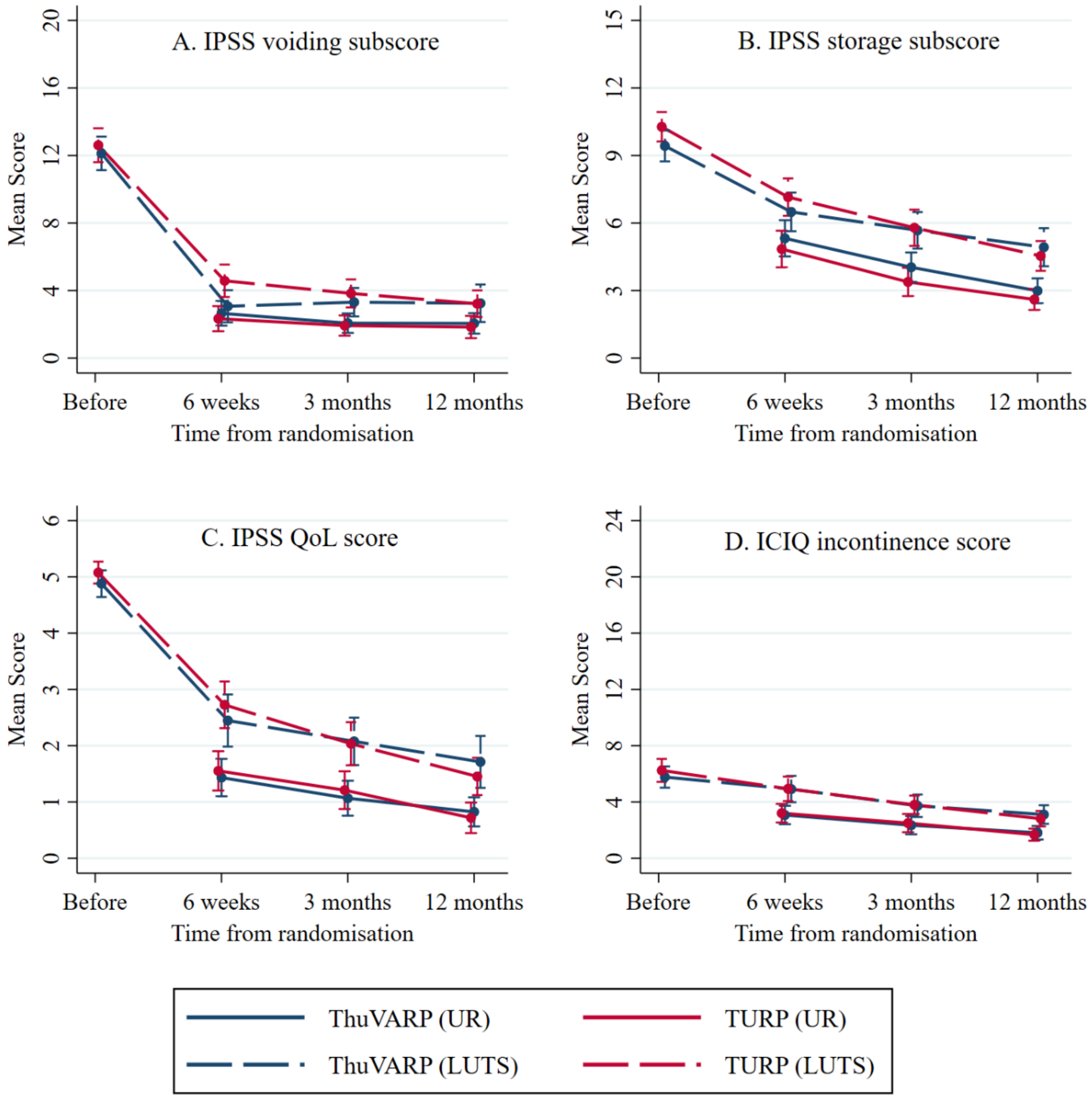

Y axis labels indicate the scale of each PROM. Data by arm is presented on an intention to treat basis. All patients who did not have an indwelling catheter at each time point are included. A few patients with UR, but without an indwelling catheter, were excluded at baseline; therefore all baseline data presented is from patients with a baseline diagnosis of LUTS rather than UR. Larger scores indicate more severe symptoms. Numbers analysed provided in supplementary table S4.

Supplementary Figure 2 (S2). Sexual function scores over time for patients presenting with LUTS or UR at baseline (ICIQ-MLUTSsex and IIEF)

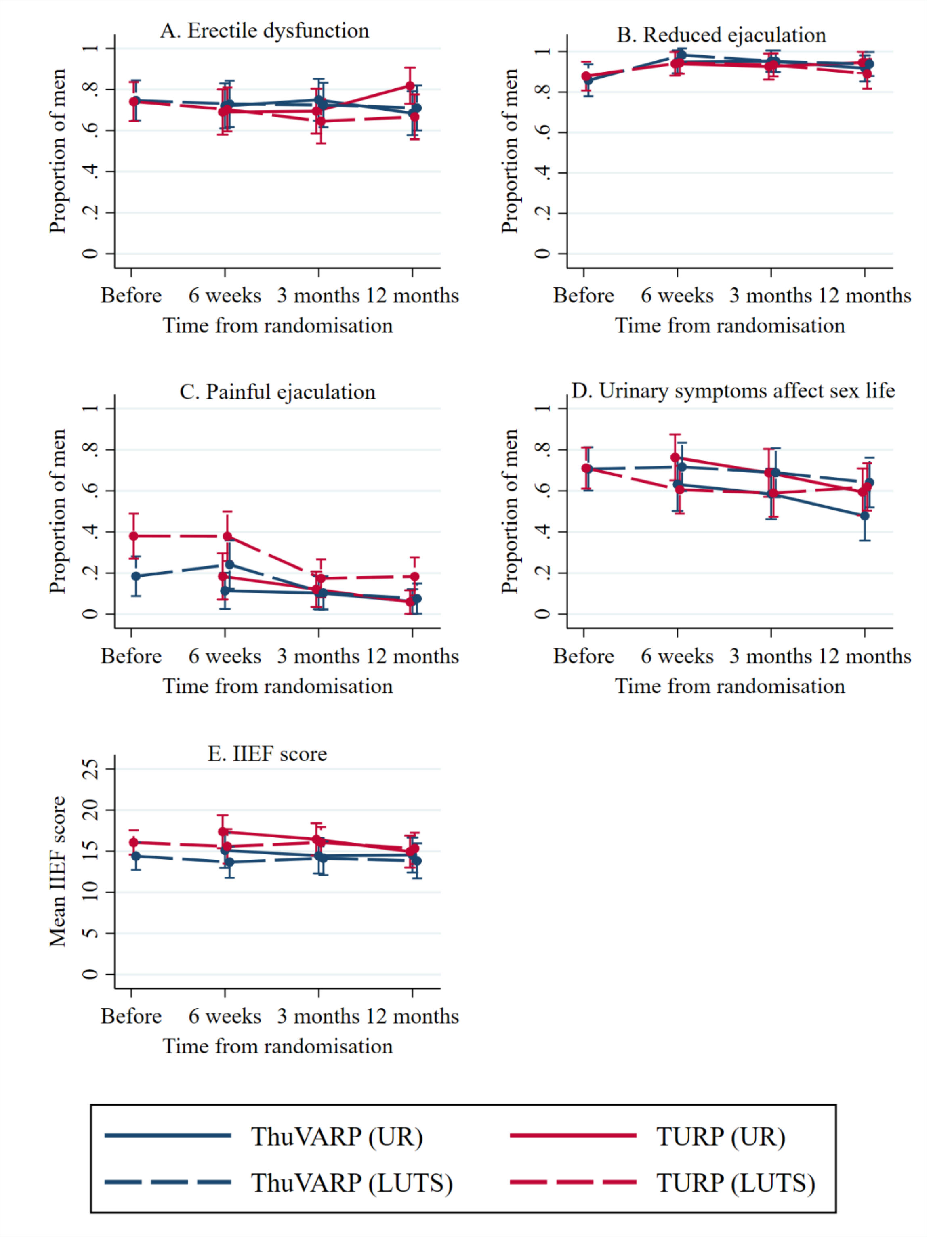

Data by arm is presented on an intention to treat basis. Where proportions are presented, men were considered to be suffering from the symptom if they reported experiencing it all, see Supplementary Table S1. IIEF score is on a scale of 5-25 with larger scores indicating more severe symptoms. All patients who did not have an indwelling catheter at each time point are included. A few patients with UR, but without an indwelling catheter, were excluded at baseline; therefore all baseline data presented is from patients with a baseline diagnosis of LUTS rather than UR. Numbers analysed provided in supplementary table S5.

Supplementary Tables

Supplementary Table 1 (S1). Dichotomous variables for ICIQ-MLUTS and MLUTS-sex

New variable	Question	Coded as 0	Coded as 1
Daytime frequency (>8)	ICIQ-MLUTS Qn 13a. How often do you pass urine during the day?	If the patient ticked '1 to 6 times' or '7 to 8 times'	If the patient ticked '9 to 10 times', '11 to 12 times' or '13 or more times'
Nocturia (>1 times per night)	ICIQ-MLUTS Qn 14a. During the night, how many times do you get up to urinate, on average?	If the patient ticked 'none' or 'one'	If the patient ticked 'two', 'three' or 'four or more'
Erections (reduced or none)	ICIQ-MLUTS-sex Qn 2a. Do you get erections?	If the patient ticked 'yes, with normal rigidity'	If the patient ticked 'yes, with reduced rigidity', 'yes, with severely reduced rigidity' or 'no, erection not possible'.
Ejaculation (reduced or none)	ICIQ-MLUTS-sex Qn 3a. Do you have an ejaculation of semen?	If the patient ticked 'yes, normal quantity'	If the patient ticked 'yes, reduced quantity', 'yes, significantly reduced quantity' or 'no ejaculation'.
Painful ejaculation	ICIQ-MLUTS-sex Qn 4a. Do you have pain or discomfort during ejaculation?	If the patient ticked 'no'	If the patient ticked 'yes, slight pain/discomfort', 'yes, moderate pain/discomfort' or 'yes, severe pain/discomfort'
Urinary symptoms affected sex life?	ICIQ MLUTS-sex Qn 5a. To what extent do you feel that your sex life has been spoilt by your urinary symptoms?	If the patient ticked 'not at all'	If the patient ticked 'a little', 'somewhat' or 'a lot'.

Supplementary Table 2 (S2). Reasons for change in treatment

Treatment received	Reason	Number of patients
Change in treatment from TURP		
Urethral stricture	Prostate reasonable size	2
Urethral stricture	Unable to access urethra	1
Bladder neck incision	Tight bladder neck	1
Change in treatment from ThuVARP		
TURP	Equipment issues (no treatment with ThuVARP)	9
TURP	Anaesthetic complications	1
TURP	No laser trained nursing staff available	1
TURP	Start time delayed, therefore proceeded with TURP	1
Conversion (TV-TP)	Equipment issues5 (converted to TURP mid procedure)	9
Conversion (TV-TP)	Very large prostate	9
Conversion (TV-TP)	Bleeding	5
Conversion (TV-TP)	Failed to progress with ThuVARP	4
Conversion (TV-TP)	To collect remaining fragments of prostate	4
Conversion (TV-TP)	Poor visibility	3
Conversion (TV-TP)	Incidental finding of tumour	1
Conversion (TV-TP)	No details found	1
Optical urethrotome	No details found	1
Prostatic embolism	Prostate too big for ThuVARP/TURP	1
Transurethral resection of bladder tumour	Risk of seeding tumour cells into prostatic urethra so no bladder outlet procedure performed	1

TV=ThuVARP, TP=TURP

Supplementary Table 3 (S3). Sensitivity Analyses: IPSS and Qmax Scores

**Variable**	**N (TV:TR)**	**ThuVARP** *Mean (SD)*	**TURP** *Mean (SD)*	**Difference in means**[a] **(95% C.I.)**
Sensitivity: IPSS Symptom Score				
ITT Complete case analysis	151:159	6.29 (6.22)	6.03 (5.21)	0.43 (-0.78, 1.64)
Per protocol[b]	114:156	5.78 (5.63)	5.99 (5.25)	-0.04 (-1.28, 1.21)
CACE analysis[c]				0.05 (-1.22, 1.32)
Removal of patients[d]	147:157	6.04 (5.84)	5.97 (5.13)	0.26 (-0.92, 1.44)
Adj. for baseline[e]	143:146	6.05 (5.78)	6.07 (5.23)	0.13 (-1.08, 1.34)
Adj. for imbalance[f]	52:67	8.00 (6.71)	7.79 (5.93)	0.28 (-1.98, 2.53)
Surgeon effects[g]	149:157	6.29 (6.26)	6.03 (5.24)	0.44 (-0.76, 1.65)
Post-hoc: Bootstrap[h]				0.43 (-0.77, 1.64)
Post-hoc: Log transformation[i]	151:159	1.67 (0.81)	1.69 (0.73)	0.00 (-0.16, 0.17)
Sensitivity: Qmax level				
ITT Complete case analysis	168:176	20.19 (12.43)	23.47 (12.82)	-3.42 (-6.10, -0.73)
Per protocol[b]	123:172	19.30 (11.01)	23.75 (12.83)	-4.61 (-7.39, -1.83)
CACE analysis[c]	123:181	19.30 (11.01)	23.71 (12.94)	-4.67 (-7.56, -1.78)
Removal of patients[d]	163:173	20.12 (12.19)	23.51 (12.87)	-3.47 (-6.16, -0.77)
Adj. for baseline[e]	155:162	19.81 (11.87)	23.39 (12.42)	-3.87 (-6.57, -1.16)
Adj. for imbalance[f]	60:80	19.93 (11.16)	23.90 (12.61)	-3.95 (-8.07, 0.17)
Surgeon effects[g]	165:175	20.28 (12.46)	23.53 (12.84)	-3.44 (-6.11, -0.78)
Post-hoc: Bootstrap[h]				-3.42 (-6.06, -0.78)
Post-hoc: Log transformation[j]	168:176	2.83 (0.61)	2.99 (0.60)	-0.17 (-0.29, -0.04)

TV=ThuVARP, TP=TURP. [a]Adjusted for centre & whether the patient had retention or LUTS at baseline. [b]Removing those who did not comply with their randomised treatment. [c]Unbiased estimates to account for 12 non-compliers in the ThuVARP arm that instead received a TURP (this does not include conversions mid procedure), resulting in 6 additions when analysing IPSS and 9 additions when analysing Qmax. [d]Patients who found out their allocation prior to completing the 12 months questionnaire. [e]Respective baseline measures for the IPSS (baseline IPSS was broken down into mild/moderate/severe and men with indwelling catheters were imputed as severe) and Qmax (men with indwelling catheters were imputed as zero). [f]Imbalances at baseline by more than 10%/0.5 SDs (painful ejaculation). [g]A mixed effects model that includes the surgeon as a random effect and centre as a fixed effect. [h]Bootstrap linear regression with 4999 replications. [i]Log transforming (natural log) an adjusted IPSS score (IPSS score + 1) with new equivalence margin of 0.92. [j]Log transforming (natural log) the Qmax with new equivalence margin of 1.39.

Supplementary Table 4 (S4). Subgroup Analyses: IPSS at 12 months

	IPSS score at 12 months				
**Variable**	**ThuVARP** *Mean(SD); n*	**TURP** *Mean(SD); n*	**Subgroup specific MD (95% C.I)[a]**	**Interaction MD (95% C.I)[b]**	**P[c]**
Subgroup analyses					
**Baseline diagnosis**					
LUTS	8.19 (7.38); 64	7.63 (5.72); 78	0.52 (-1.63, 2.67)	-	0.888
Urinary retention	4.90 (4.80); 87	4.49 (4.16); 81	0.32 (-1.03, 1.68)	-0.17 (-2.61, 2.27)	
**Age**					
<70	6.83 (7.04); 75	6.27 (5.59); 90	1.00 (-0.83, 2.83)	-	0.519
≥70	5.76 (5.30); 76	5.72 (4.70); 69	-0.07 (-1.68, 1.53)	-0.79 (-3.23, 1.66)	
**Peri-operative prostate size**					
Small (<40g)	6.21 (5.39); 68	6.38 (5.69); 63	-0.18 (-2.10, 1.74)	-	0.614
Medium (40-60g)	6.68 (6.63); 41	5.72 (5.34); 53	0.54 (-1.64, 2.72)	0.90 (-2.00, 3.81)	
Large (60-80g)	5.40 (7.22); 20	4.65 (3.33); 17	-0.25 (-3.97, 3.47)	1.20 (-2.85, 5.24)	
Very large (>80g)	6.67 (7.35); 12	5.50 (3.07); 8	3.76 (-3.08, 10.59)	2.61 (-2.71. 7.93)	
**Comorbidities at baseline**					
With	6.27 (5.56); 67	6.77 (5.39); 64	-0.60 (-2.43, 1.23)	-	0.202
Without	6.31 (6.74); 84	5.54 (5.06); 95	0.92 (-0.73, 2.56)	-1.58 (-4.05, 0.89)	

MD refers to difference in means, [a]Linear regression model adjusting for centre and baseline diagnosis where appropriate, [b]The coefficient for the interaction term, [c]Likelihood ratio test comparing models including/excluding the interaction term.

Supplementary Table 5 (S5). Subgroup Analyses: Qmax at 12 months

	Qmax at 12 months				
**Variable**	**ThuVARP** *Mean(SD); n*	**TURP** *Mean(SD); n*	**Subgroup specific MD (95% C.I)[a]**	**Interaction MD (95% C.I)[b]**	**P[c]**
Subgroup analyses					
**Baseline diagnosis**					
LUTS	18.68 (10.11); 78	23.81 (12.36); 95	-5.11 (-8.53, -1.69)	-	0.189
Urinary retention	21.51 (14.06); 90	23.07 (13.42); 81	-1.56 (-5.77, 2.65)	3.54 (-1.84, 8.91)	
**Age**					
<70	22.33 (13.41); 85	26.69 (12.62); 99	-5.26 (-9.10, -1.43)	-	0.114
≥70	18.00 (11.00); 83	19.33 (11.93); 77	-1.06 (-4.60, 2.47)	4.17 (-1.09, 9.43)	
**Peri-operative prostate size**					
Small (<40g)	18.18 (9.87); 78	24.08 (13.13); 73	-6.17 (-9.95, -2.39)	-	0.774
Medium (40-60g)	22.60 (12.69); 42	23.70 (12.85); 57	-1.09 (-6.34, 4.15)	4.84 (-1.74, 11.42)	
Large (60-80g)	20.25 (12.35); 21	23.19 (12.04); 18	0.64 (-8.23, 9.50)	2.83 (-6.36, 12.02)	
Very large (>80g)	20.43 (20.08); 15	24.68 (18.98); 9	-1.38 (-22.52, 19.76)	1.64 (-9.84, 13.13)	
**Comorbidities at baseline**					
With	19.17 (12.33); 75	21.02 (11.44); 76	-1.80 (-5.63, 2.02)	-	0.307
Without	21.02 (12.52); 93	25.33 (13.55); 100	-4.91 (-8.67, -1.15)	2.79 (-2.66, 8.25)	

MD refers to difference in means. [a]Linear regression model adjusting for centre and baseline diagnosis where appropriate. [b]The coefficient for the interaction term. [c]Likelihood ratio test comparing models including/excluding the interaction term.

Supplementary Table 6 (S6). Number of men included at each time point in Figures 2a and 2b.

	Baseline	6 weeks	3 months	12 months
Figure 2a. IPSS total score				
ThuVARP (UR)	0	81	84	87
TURP (UR)	0	76	78	81
ThuVARP (LUTS)	79	71	74	64
TURP (LUTS)	83	78	79	78
Figure 2b. Qmax (flow rate)				
ThuVARP (UR)	0	Not measured	89	90
TURP (UR)	0	Not measured	84	81
ThuVARP (LUTS)	82	Not measured	82	78
TURP (LUTS)	91	Not measured	92	95

Supplementary Table 7 (S7). Peri-operative surgical outcomes[a]

Variable	ThuVARP	TURP
	**n (%)**	**n (%)**
Peri-operative complications		
Anaesthetic complications	8/203 (4%)	4/204 (2%)
Bleeding requiring Hb measurement	4/203 (2%)	3/204 (1%)
Blood transfusion	0/203 (0%)	1/204 (<1%)
TUR syndrome	0/203 (0%)	0/204 (0%)
Perforation/extravation	4/203 (2%)	3/204 (1%)
Catheter misplacement	1/203 (<1%)	0/204 (0%)

[a]*Occuring in theatre or during the recovery period*

Supplementary Table 8 (S8). Numbers analysed for figure S1.

	Baseline	6 weeks	3 months	12 months
**A. IPSS voiding subscore**				
ThuVARP (UR)	0	86	89	89
TURP (UR)	0	81	81	86
ThuVARP (LUTS)	80	73	77	69
TURP (LUTS)	87	83	83	82
**B. IPSS storage subscore**				
ThuVARP (UR)	0	84	85	90
TURP (UR)	0	77	81	86
ThuVARP (LUTS)	82	73	77	67
TURP (LUTS)	87	79	81	80
**C. IPSS score**				
ThuVARP (UR)	0	81	84	87
TURP (UR)	0	76	78	81
ThuVARP (LUTS)	79	71	74	64
TURP (LUTS)	83	78	79	78
**D. IPSS QoL**				
ThuVARP (UR)	0	90	90	91
TURP (UR)	0	85	81	89
ThuVARP (LUTS)	83	76	78	73
TURP (LUTS)	91	84	86	86
**E. ICIQ incontinence**				
ThuVARP (UR)	0	81	86	90
TURP (UR)	0	79	80	89
ThuVARP (LUTS)	83	73	78	75
TURP (LUTS)	90	76	84	86

Supplementary Table 9 (S9). Numbers analysed for figure S2.

	Baseline	6 weeks	3 months	12 months
**A. Erectile dysfunction**				
ThuVARP (UR)	0	68	72	76
TURP (UR)	0	71	72	77
ThuVARP (LUTS)	79	63	69	69
TURP (LUTS)	85	74	79	75
**B. Reduced ejaculation**				
ThuVARP (UR)	0	60	64	73
TURP (UR)	0	67	68	75
ThuVARP (LUTS)	78	61	63	66
TURP (LUTS)	83	73	77	73
**C. Painful ejaculation**				
ThuVARP (UR)	0	53	58	65
TURP (UR)	0	49	58	68
ThuVARP (LUTS)	65	54	58	53
TURP (LUTS)	79	66	69	71
**D. Symptoms affect sex life**				
ThuVARP (UR)	0	57	65	69
TURP (UR)	0	59	64	74
ThuVARP (LUTS)	75	60	61	64
TURP (LUTS)	83	71	73	71
**E. IIEF score**				
ThuVARP (UR)	0	41	46	52
TURP (UR)	0	46	53	61
ThuVARP (LUTS)	61	48	51	48
TURP (LUTS)	68	49	56	57

Supplementary Table 10 (S10). Cross tabulation of IIEF severity at baseline with 12-months post-surgery

	**IIEF at 12 months**						
**IIEF at baseline**	**None**	**Mild**	**Mild to moderate**	**Moderate**	**Severe**	**Missing**	**Total**
None	19	4	1	1	0	5	30
Mild	4	8	5	2	3	14	36
Mild to moderate	1	4	10	2	4	11	32
Moderate	0	2	2	2	6	6	18
Severe	0	1	1	1	12	8	23
Missing	30	27	16	13	37	148	271
Total	54	46	35	21	62	192	410

Responses for men with an indwelling catheter were coded as missing.

Supplementary Table 11 (S11). Pathological findings (Intention to treat)

**Variable**	**N (TV:TR)**	**ThuVARP n(%)/*Median(IQR)***	**TURP n(%)/*Median(IQR)***	**Difference (95% C.I.)**	**P value**
Prostate histology					
Resection weight (g)	149:162	*7.0 (2.0, 15.0)*	*20.0 (11.0, 35.0)*	-15.4[a] (-19.3, -11.5)	<0.001
Benign	193:193	182 (94%)	166 (86%)		
Prostate cancer	193:193	10 (5%)	25 (13%)	0.35[b] (0.16, 0.75)	0.007
High grade PIN	193:193	1 (1%)	2 (1%)		

TV=ThuVARP, TR=TURP, [a]Linear regression adjusted for centre & whether the patient had retention or LUTS at baseline (results were very similar when using non-parametric tests) [b]Logistic regression, comparing PCa detection with benign histology (excluding HGPIN), adjusted for centre & whether the patient had retention or LUTS at baseline
